# Establishment of *Agrobacterium*-mediated genetic transformation and application of CRISPR/Cas9 genome-editing system to *Brassica rapa* var. *rapa*

**DOI:** 10.1186/s13007-022-00931-w

**Published:** 2022-08-06

**Authors:** Yuanyuan Liu, Li Zhang, Cheng Li, Yunqiang Yang, Yuanwen Duan, Yongping Yang, Xudong Sun

**Affiliations:** 1 Germplasm Bank of Wild Species, Kunming Institute of Botany, Chinese Academy of Sciences, Kunming, 650201 China; 2 Institute of Tibetan Plateau Research at Kunming, Kunming Institute of Botany, Chinese Academy of Sciences, Kunming, 650201 China; 3 University of Chinese Academy of Sciences, Beijing, China

**Keywords:** Turnip, *Brassica rapa* var. *rapa*, *BrrWUSa*, Genetic transformation, Genome editing

## Abstract

**Background:**

Genome editing is essential for crop molecular breeding. However, gene editing in turnip (*Brassica rapa* var. *rapa*) have not been reported owing to the very low transformation efficiency.

**Results:**

In this study, we established a transformation procedure involving chemical-inducible activation of the *BrrWUSa* gene, which resulted in high transformation frequencies of turnip. Estradiol-inducible *BrrWUSa* transgenic plants were fertile and showed no obvious developmental defects. Furthermore, we used CRISPR/Cas9 gene-editing technology to edit *BrrTCP4b* and generated 20 *BrrTCP4b*-edited seedlings with an increase in leaf trichome number.

**Conclusion:**

The results demonstrate that *BrrWUSa* improves the regeneration efficiency in turnip. The transformation procedure represents a promising strategy to improve genetic transformation and for functional characterization of genes in turnip.

**Supplementary Information:**

The online version contains supplementary material available at 10.1186/s13007-022-00931-w.

## Background

The development and optimization of genome-editing tools has enabled precise genome modifications and research on gene function has been boosted. Owing to their accuracy, efficiency, and cost-effectiveness, CRISPR/Cas systems have been widely applied to diverse organisms [1]. *Agrobacterium*-mediated genetic transformation is an important means to achieve efficient gene delivery and editing for CRISPR/Cas systems. However, low efficiencies in plant regeneration and dependence on a limited number of transformable genotypes hinder gene-editing tool implementation for crop improvement [2, 3]. Thus, improvement of regeneration efficiency remains an important research focus.

Research on development regulators is crucial to improve plant regeneration, given the key functions of development regulators in cell differentiation and morphogenesis. A suite of development regulators that promote plant regeneration in tissue culture has been reported, such as WUSCHEL (WUS) [4], LEAFY COTYLEDON 1 (LEC1) [5], BABY BOOM (BBM) [6], and AGAMOUSLIKE 15 (AGL15) [7]. Overexpression of such regulators can promote regeneration of various transformation-recalcitrant genotypes and species. WUS, which encode a homeodomain transcription factor, has been exploited to promote cell differentiation and plant regeneration, and plays a critical role in stem cell fate determination and maintenance in the shoot apical meristem of higher plants [8]. For example, overexpression of *A. thaliana WUS* induces initiation of stem cells in vegetative tissues, which can differentiate into somatic embryos in the absence of exogenous plant hormones [4]. Recent study shows that overexpression of the wheat gene *TaWOX5* significantly increases wheat transformation efficiency [9]. Expression of the maize development regulators BBM and WUS2 results in high transformation frequencies of maize and other transformation-recalcitrant monocotyledonous species [6].

Turnip (*Brassica rapa* var. *rapa*) is used mainly as a vegetable and for fodder. The antihypoxic activity of turnip provides the potential to prevent altitude stress [10, 11]. Therefore, an improved understanding of the bioactivities is of medicinal importance. However, further research on gene function of turnip has been greatly hampered by the low efficiency of transformation and shoot regeneration. Establishment of a high-efficiency *Agrobacterium*-mediated genetic transformation method for turnip requires optimization of callus proliferation and shoot regeneration during tissue culture.

In this study, we show that inducible expression *BrrWUSa* promotes callus formation and shoot regeneration, and establish a high-efficiency *Agrobacterium*-mediated genetic transformation system for turnip. Furthermore, we successfully generated *BrrTCP4b*-edited plants using the CRISPR/Cas9 system. This research achieves breakthroughs in the genetic transformation and gene editing in turnip, providing a technological foundation to gain insight into gene function in turnip in future studies.

## Results

### *BrrWUSa* promotes callus formation and shoot regeneration in turnip

**Fig. 1:**
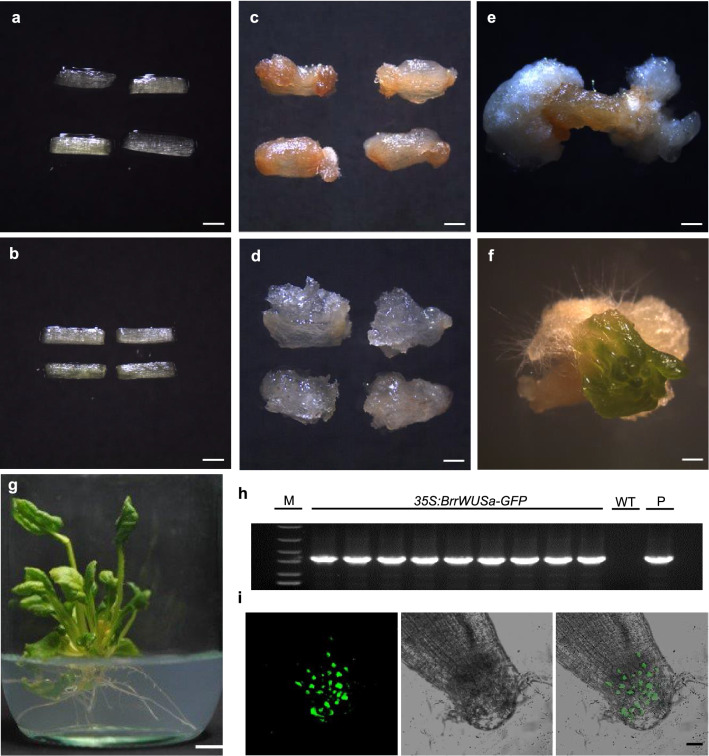
Effect of *BrrWUSa* on shoot regeneration frequency. Hypocotyl segments co-cultured with *35S:GFP* (**a**) or *35S:BrrWUSa-GFP* (**b**). **c**, **d** Callus formation on selection medium with kanamycin and hormone after 2 weeks in vitro culture. **e**, **f** Representative transformation showing a higher frequency of transgenic *BrrWUSa* plants and the empty vector controls. **g** Transgenic seedling on root induction medium. **h** Transgenic-specific PCR product amplified with primers GFP-F and BrrWUSa-R. M, DNA ladder marker DL2502. **i** Green fluorescence observation in the root tips of *35S:BrrWUSa-GFP* transgenic plants. Scale bars = 1 mm in **a**–**f**, 1 cm in **g** and 30 μm in **i**

Although tissue culture of turnip readily produces callus, the shoot regeneration has proved to be a bottleneck. To enhance shoot formation, we aimed to reprogram plant meristems by expressing a development regulator. Three *WUS* homolog genes were identified in the turnip genome, and based on its higher expression levels in shoot apical meristem (SAM), *BrrWUSa* was selected for assessment of regeneration frequencies (Additional file 1: Fig. S1). The hypocotyls of 4-day-old turnip seedlings were cut into 3–5 mm segments as explants. The explants were inoculated with *Agrobacterium* harboring *35S:GFP* (as a control) or *35S:BrrWUSa-GFP* for 15 min, and then transferred to callus-induction medium (Fig. 1a, b). The explants were cultured for 2 days in darkness condition to increase the infection efficiency, then were transferred to long-day condition (16-h light/8-h darkness, 22 ℃). After 2 weeks, explants of control hypocotyl segments and *35S:BrrWUSa-GFP* hypocotyl segments gave rise to callus (Fig. 1c, d). After 4 weeks, no shoots regenerated form from control hypocotyl segments (Fig. 1e; Table 1), whereas shoots regenerated from hypocotyl segments inoculated with *35S:BrrWUSa-GFP* (Fig. 1f). Hypocotyls transformed with *35S:BrrWUSa* showed 13% regeneration efficiency, whereas no shoots regenerated in the control (Table 1).The regenerated shoots were transferred to rooting medium, and whole plantlets formed after 30 days (Fig. 1g). The transformants were verified by PCR and confocal laser scanning microscopy. Using gene-specific primers containing the upstream sequence of *GFP* gene (F) and downstream of *BrrWUSa* (R), the amplified band was detected in all regeneration lines, but not in wild type (Fig. 1h). Confocal laser scanning microscopy observed fluorescence in the nucleus of root cells in *35S:BrrWUSa-GFP* transgenic plants, which indicate the successful expression of BrrWUSa-GFP fusion protein at the root apical meristem and verifies the transformants (Fig. 1i). These results suggested that *BrrWUSa* could promote shoot regeneration in turnip.

**Table 1:** Representative transformation efficiency of kanamycin-resistant callus and regenerated shoots in the presence of *BrrWUSa*

*Agrobacterium*	No. of explants	Resistant calli induction rate (%)	Resistant shoot induction rate (%)
*35S:GFP*	80	10	0
*35S:BrrWUSa-GFP*	80	83	13

### Inducible expression of *BrrWUSa* improves shoot regeneration and generates fertile plants

**Fig. 2:**
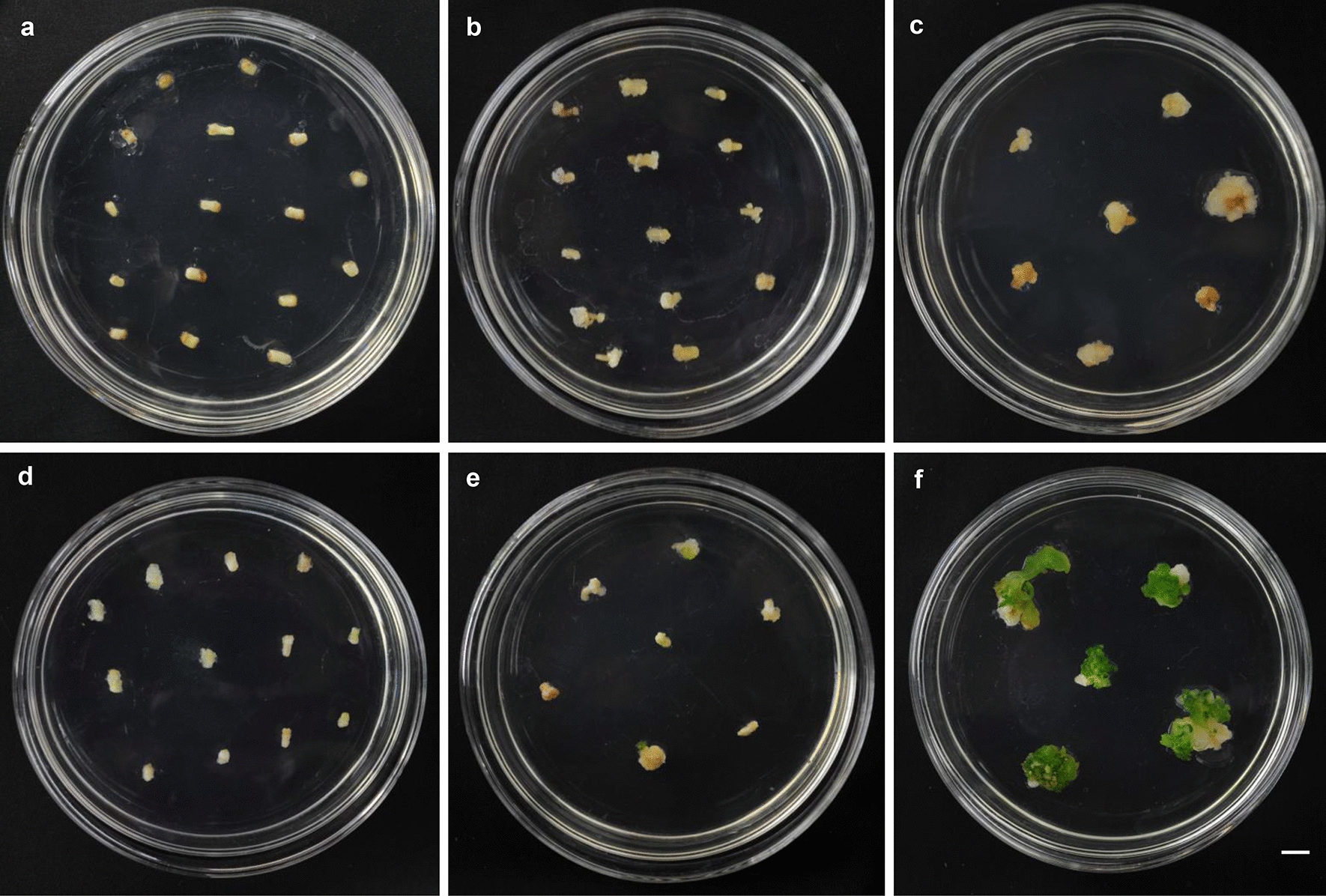
*pER8*-*BrrWUSa* induces regeneration shoots in the presence of estrogen. **a**–**c** No regeneration shoots formed in the absence of estrogen. **d**–**f** High efficiency of regeneration shoot formed in the presence of estrogen. Scale bar = 0.5 cm.

**Fig. 3:**
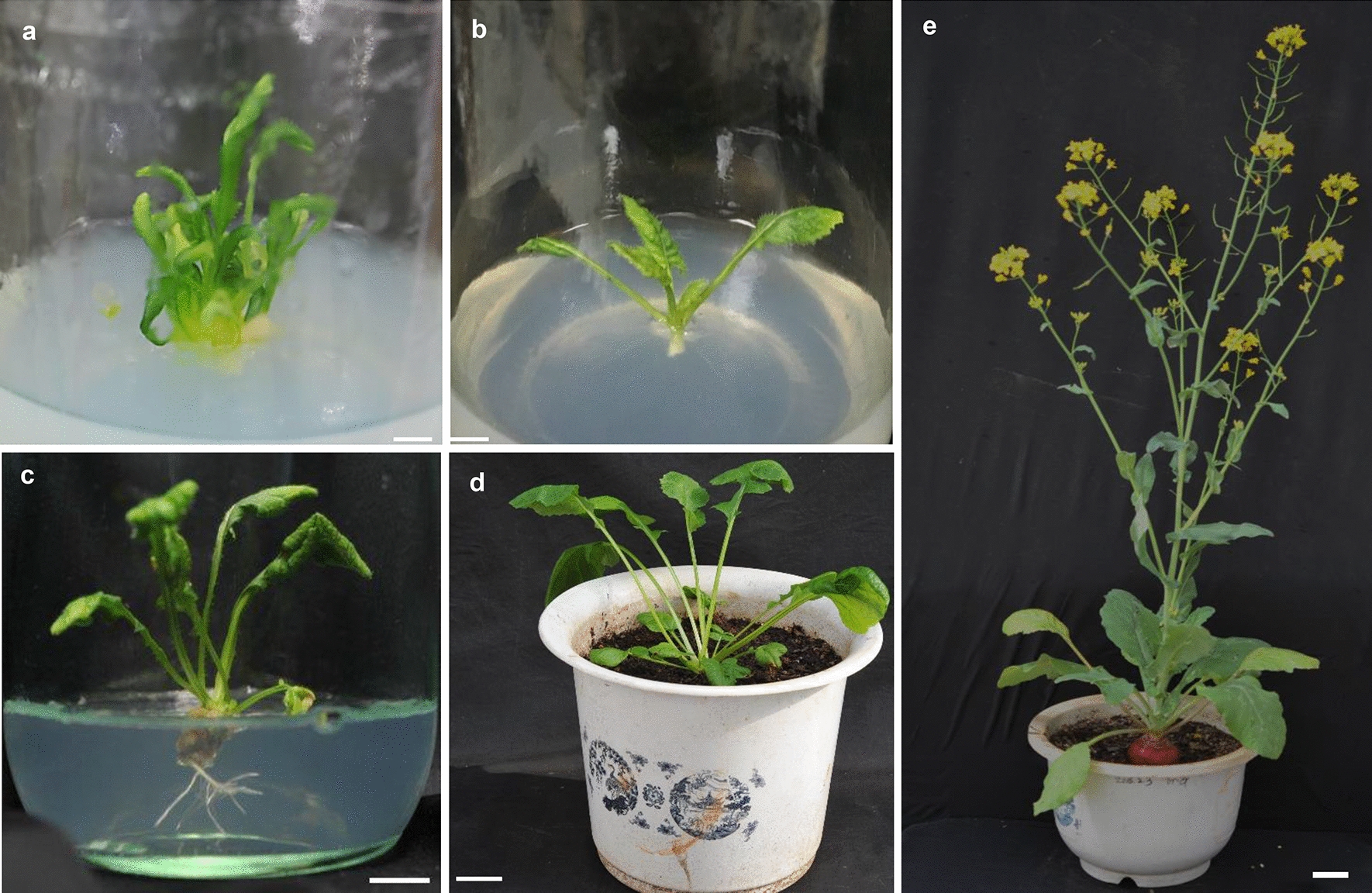
Phenotypes of *pER8-BrrWUSa* transgenic plants. The *pER8-BrrWUSa* transgenic plants were normal and fertile. Scale bars = 1 cm in **a**–**c**, and 3 cm in **d** and **e**

Overexpression of *BrrWUSa* in turnip resulted in sterile plants with abnormal leaf phenotype (Additional file 1: Fig. S2). To avoid the harmful effects of *BrrWUSa*, *BrrWUSa* was subcloned into the pER8 [12]. Turnip hypocotyl explants transformed with *Agrobacterium* carrying *pER8-BrrWUSa* were cultured on callus-induction medium supplemented with or without estradiol (Fig. 2a, d). After 4 weeks co-culture, explants on both medium gave rise to callus (Fig. 2b, e). The result show that explants untreated with estradiol were unable to generate shoots (Fig. 2c), the callus produced shoots after incubation for 4 weeks on medium supplemented with estradiol (Fig. 2e, f). The regenerated shoots were transferred to soil after incubation in root-induction medium (Murashige and Skoog medium) (Fig. 3). The regenerated shoots developed into complete plants with estradiol treatment, and transgenic plants were fertile without obvious developmental defects (Fig. 3e). These results indicated that inducible expression of *BrrWUSa* in turnip increased the efficiency of plant regeneration.

### Characterization of *BrrTCP4b*-targeted editing in turnip transformants

**Fig. 4:**
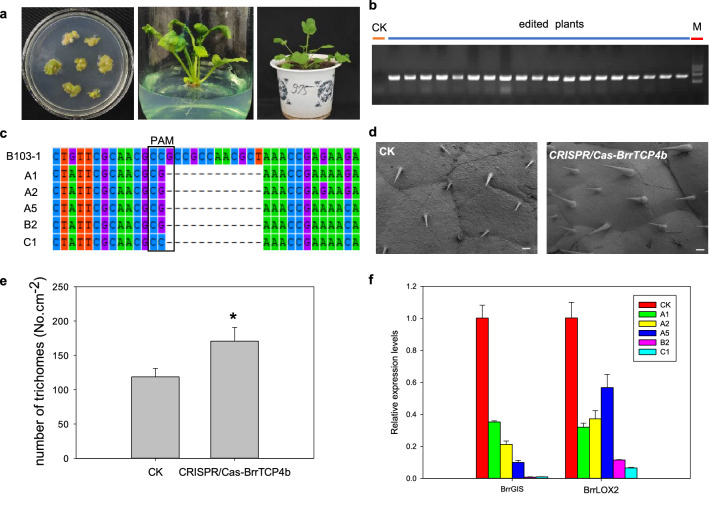
High frequency of genome-edited plants using CRISPR-Cas9 technology in *pER8-BrrWUSa* transgenic plant. **a** Gene-edited regeneration plants germinated from the callus. **b** Transgenic-specific PCR product amplified with primers Cas9-F and Cas9-R. CK, mock control; edited plants; M, DNA ladder marker DL2502. **c** Sequencing results of indels at the desired place. Twenty plants carried 5 different mutations including A1, A2, A5, B2, C1. B103-1 represented sequences of wild type. (**d**, **e**) Edited T_0_ plants showing an increased number trichomes. Asterisk (*) above the bars represents significant difference according to Student’s *t*-test analysis. Scale bars = 100 μm in **d**. **f** qRT-PCR analysis of *BrrTCP4b* targeted genes. The expression levels of all genes in the CK are set to 1. *Tubulin* was amplified as a constitutive control. Relative expression level of each gene was calculated by normalizing to the value in CK plants. Relative expression levels were calculated using 2^−ΔΔCt^ method. Error bars represent Standard Deviation of triplicate experiments. Letters above the bars indicate significant difference according to one-way ANOVA analysis at P < 0.05

To demonstrate the utility of plant transformation to generate genome-edited plants, turnip *TEOSINTE BRANCHED1*/*CYCLOIDEA*/*PCF 4b* (*BrrTCP4b*) was selected for genome editing. The constructs expressing gRNA targeting the *BrrTCP4b* gene were transformed into *pER8-BrrWUSa* transformant hypocotyls and incubated in the dark for 72 h. After incubation for 2 weeks on callus-induction medium supplemented with 2 μM estradiol, all explants developed embryogenic calli and were then transferred to shoot-induction medium supplemented with 2 μM estradiol and 20 mg/L kanamycin. Then we transferred the regenerated shoots to root-induction medium supplemented with 20 mg/L kanamycin. All shoots formed complete plants and were fertile (Fig. 4a). We extracted genomic DNA and examined mutations or deletions within the targeted region (Fig. 4b, c). Sequencing of the cloned PCR products verified successful deletions at the expected positions. Twenty gene-edited T_0_ plants were transferred to soil. Phenotype analysis revealed that the number of trichomes in *CRISPR-Cas9-BrrTCP4b* plants was approximately 150% greater than the pER8-WUSa mock plant (Fig. 4d, e). TCP4 directly activate the expression of *GLABROUS INFLORESCENCE STEMS* (*GIS*) and *lipoxygenase2* (*LOX2*) to regulate the formation of trichome [13, 14]. Therefore, quantitative RT-PCR assays were conducted to detect the expression levels of targeted genes. The expression levels of *BrrGIS* and *BrrLOX2* in *CRISPR-Cas9-BrrTCP4b* plants were significantly decreased compared with those of the mock plant (Fig. 4f). These results demonstrated that *BrrWUSa* may significantly improve the regeneration efficiency and CRISPR/Cas9-mediated genome editing in turnip.

## Discussion

Genome editing shows strong potential for crop improvement, but its application is restricted by low plant regeneration frequencies and genotype dependence [2, 15]. In recent years, efforts to improve plant regeneration frequencies have continued. Overexpression of plant development regulators greatly increases regeneration and transformation frequencies. Research on genetic transformation of turnip, a valuable cruciferous crop, is hardly a blank.

Given that shoot regeneration efficiency is essentially genotype-dependent, we estimated the genotype effect of more than one hundred turnip landraces. However, plant regeneration was unsuccessful for all landraces. Studies have reported improvements in the efficiency of plant regeneration from tissue culture by overexpression of plant development regulators, such as *WUS* and *BBM* [4, 6, 16] and *GRF*-*GIF* chimeras [2, 15, 17]. Recent studies indicate overexpression of *TaWOX5* is an efficient way to help wheat overcomes genotype dependency and promote genetic transformation [9]. To increase the regeneration efficiency of turnip calli, we cloned *BrrWUSa*, which showed highest expression levels among *WUS* homologs in turnip. Our study indicate that *BrrWUSa* promotes callus formation and shoot regeneration in turnip.

However, the breakthrough and improvement of both regeneration frequency and genome-editing efficiency is confined to overexpression of the specific plant development regulators which can reprogram somatic cells to embryogenic cell [4, 6, 16]. Based on the high regeneration efficiency of *BrrWUSa* overexpression, we transfer the target gene into the transgenic plants of *BrrWUSa* and to assess the genome-editing efficiency. Besides, to avoid sterility and obvious developmental defects in transformants caused by *BrrWUSa* overexpression, we generated the estrogen-inducible construct *pER8-BrrWUSa*. Hypocotyl segments transformed with *pER8-BrrWUSa* successfully generated shoots under estrogen treatment. The regenerated shoots developed into complete plants and were fertile without obvious developmental defects observed. Taking advantage of this transformation procedure, CRISPR/Cas-mediated genome editing was conducted. Twenty T_0_ plants all showed clear DNA deletion and mutation. *TCP4* plays a negative effect on trichome differentiation and suppress trichome branching in Arabidopsis leaves and inflorescence stem [13]. Our research showed the number of trichomes in *CRISPR-Cas9-gRNA-BrrTCP4b* plants increased significantly in gene-edited compared with mock plant, and the relative expression levels of downstream target genes of *BrrTCP4* significantly decrease. These results demonstrated that *BrrWUSa* may enhance CRISPR/Cas9-mediated genome editing in turnip.

In summary, we successfully established a stable genetic transformation and gene-editing procedure for turnip. Inducible expression of *BrrWUSa* significantly increased the regeneration efficiency and generated a large number of fertile gene-edited plants. The method may play an important role in functional characterization of genes for turnip and provides a powerful tool for future improvement of turnip by CRISPR/Cas-mediated gene editing.

## Materials and methods

*Brassica rapa* var*. rapa* Landrace KTRG-B-103 (Xichang, Sichuan, China) was selected owing to its high differentiation rate determined in a preliminary experiment. KTRG-B-103 homozygous F1 seeds were harvested after self-pollination. The seeds were sterilized in petri dishes containing 1% sodium hypochlorite solution for 10 min and rinsed five times with sterilized deionized water. The sterilized seeds were sown on half-strength MS medium (pH 5.8) supplemented with 3% (w/v) sucrose and 0.5% (w/v) Phytagel. The seeds germinated for 2 days in the darkness, and then transferred to long-day condition (16-h light/8-h darkness, 22 ℃) and geminated for 2 days. Subsequently, hypocotyl explants (about 3–5 mm in length) were prepared with a scalpel and infected by *Agrobacterium*.

### Plasmid constructions

The full-length coding DNA sequence of *BrrWUSa* was cloned in accordance with the method of Li et al. [18] and subcloned into the *pRI101-GFP* vector using the ClonExpress II One Step Cloning Kit (Vazyme Biotech) (Additional file 1: Fig. S3a). For cloning, the primers F (5ʹ-*GCAGCGGCCGTCGACATGGAGCAACCGCAACATCA*-3ʹ) and R (5ʹ-GTTGATTCAGAATTC TTAATCCGGTGAGACGCCTG-3ʹ) were used.

The *pER8-BrrWUS* plasmid was reconstructed in accordance with the method of Guo et al., [19]. *BrrWUSa* was subcloned into pENTR™/D-TOPO (Invitrogen) using the primers F (5ʹ-CACCatggagcaaccgcaacatca-3ʹ) and R (5ʹ-atccggtgagacgcctg-3ʹ) for the entry clone. The *pENTR-BrrWUSa* plasmid was chosen to proceed to the LR reaction with pER8-GATEWAY-3Flag and generated the *pER8-BrrWUSa* plasmid (Additional file 1: Fig. S3b). Then BrrTCP4b target sequence was fused into CRISPR/Cas9 vector to generate the genome-editing construct (Additional file 1: Fig. S3c).

### *Agrobacterium*-mediated transformation

The reconstructed plasmids *pRI101-BrrWUSa-GFP* and *pER8-BrrWUSa* were transferred into *A*. *tumefactions* strain EHA105 by electroporation, respectively. Positive transformants were identified by PCR, then incubated for two days in 50 mL liquid YEB medium at the ratio of 1:50. The culture was centrifuged at 5000×*g* for 10 min and adjusted to OD_600_ = 0.3 for the inoculation buffer (half-strength MS supplemented with 100 mM acetosyringone). The explants were infected for 15 min and then transferred to the MS medium. After co-cultivation for 3 days in the dark, the explants were rinsed five times with sterile water supplemented with 500 mg/L cefalexin and transferred to MS medium supplemented with 6-benzylaminopurine (4.0 mg/L), naphthaleneacetic acid (1.5 mg/L), and Timentin (100 mg/L). The medium was renewed every 2 weeks. The regenerated shoots were excised and transferred to root induction medium (MS medium).

### Gene expression analysis

Total RNA was extracted using the Eastep^®^ Super Total RNA Extraction Kit (Promega, Beijing, China) according to the manufacturer's instructions. One microgram total RNA was used for the first-strand cDNA synthesis in 20µL reaction volumes containing GoScript™ Reverse Transcriptase (Promega). 2 µL cDNA, 6 µL distilled water, 10 µL FastStart Universal SYBR Green Master Mix (ROX), and gene-specific primers was mixed to generate 20 μL qPCR reaction mixtures. The Step One Plus Real-Time PCR System (Applied Biosystems) was used for the PCR program, which comprised one cycle (50 °C, 2 min), one cycle (95 °C, 10 min), 40 cycles (95 ℃ for 5 s, 60 ℃ for 15 s, 72 ℃ for 5 s), and one cycle (72 ℃, 10 min). Three independent biological replicates were performed. *Tubulin* was amplified as a constitutive control. Relative expression levels were calculated using 2^−ΔΔCt^ method. The primers used for quantitative RT-PCR are listed in Additional file 1: Table S1.

### Fluorescence microscopy

Root tips from transgenic plants were excised and GFP fluorescence was observed with an OLYMPUS confocal microscope equipped with a 480/530 nm excitation filter.

### Cryo-scanning electron microscopy

Cryo-scanning electron microscopy was used to observe the trichomes on the leaf. A section of leaf (approximately 3- × 3-mm) along the length of the lamina and midway between the margin and the mid-vein was fixed in a custom sample holder and submerged in liquid nitrogen for 2 min. A cryo-transfer shuttle Quorum pp3010t was used to transfer the sample to a precooled chamber under vacuum for coating. The samples were then observed at an accelerating voltage of 7.0 kV with a scanning electron microscopy (ZEISS Sigma 300, Zeiss, Oberkochen, Germany) with a cryogenic stage maintained at – 140 ℃.

### Molecular analysis of CRISPR/Cas9 target sites

Genomic DNA was extracted from seedlings according to CTAB method. The primers BrrTCP4bF and BrrTCP4bR were used to amplify *BrrTCP4b*. The PCR products were sequenced and aligned with the MEGA11 [20].

### Statistical analysis

Statistical analyses of the experimental data were performed using single-factor analysis of variance or Student’s *t*-test implemented in SPSS (Version 17.0).

### Accession numbers

Sequence data for most genes studied in this article can be found in the National Center for Biotechnology Information under the following accession numbers: *BrrTCP4b* (KY608005); *BrrWUSa* (MN481054); *BrrGIS* (ON887158) and *BrrLOX2* (ON887159).

## Supplementary Information

**Additional file1: Fig. S1.** Relative expression levels of *BrrWUS*, *BrrWUSa*, and *BrrWUSb* in turnip. **Fig. S2.** Phenotype of *35S:BrrWUSa* transgenic plants. **Fig. S3.** Schematic structure of three vectors. **Fig. S4.** Raw result for Fig. 1h. **Fig. S5.** Raw result of Fig. 4b. **Table S1.** Primers used in this study.

## Data Availability

The datasets used and/or analyzed during the current study are available from the corresponding author on reasonable request.
