# Supplementary material for: Establishment of Agrobacterium-mediated genetic transformation and application of CRISPR/Cas9 genome-editing system to Brassica rapa var. rapa
Source: Plant Methods. 2022 Aug 6;18:98. doi: 10.1186/s13007-022-00931-w (PMC9356411; doi:10.1186/s13007-022-00931-w)
Supplement: Supplementary file 1 — Additional file1: Fig. S1. Relative expression levels of BrrWUS, BrrWUSa, and BrrWUSb in turnip. Fig. S2. Phenotype of 35S:BrrWUSa transgenic plants. Fig. S3. Schematic structure of three vectors. Fig. S4. Raw result for Fig. 1h. Fig. S5. Raw result of Fig. 4b. Table S1. Primers used in this study. [file 13007_2022_931_MOESM1_ESM.pptx]

## Slide 1
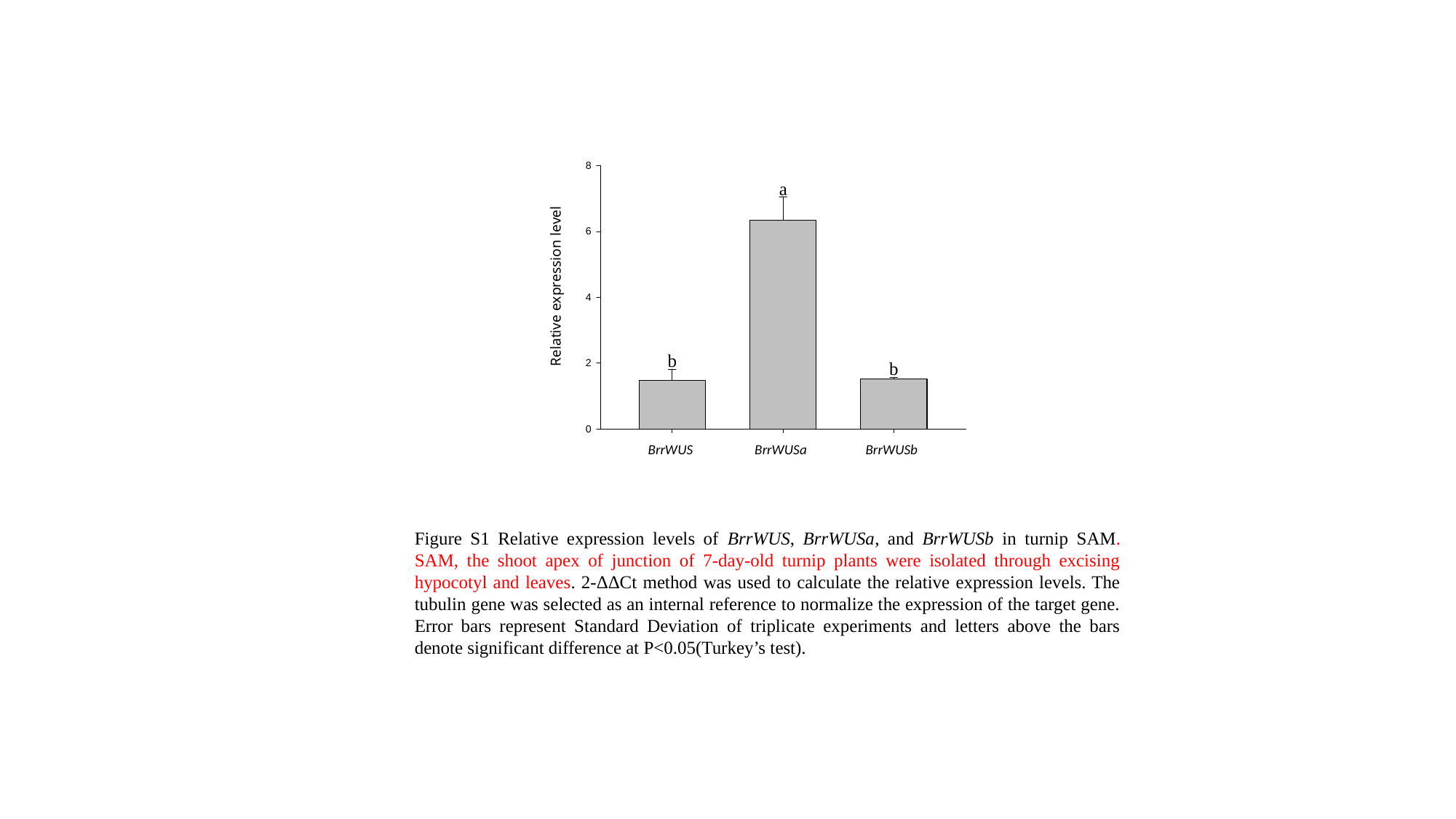

Relative expression level
 BrrWUS BrrWUSa BrrWUSb
a
b
b
Figure S1 Relative expression levels of BrrWUS, BrrWUSa, and BrrWUSb in turnip SAM. SAM, the shoot apex of junction of 7-day-old turnip plants were isolated through excising hypocotyl and leaves. 2-ΔΔCt method was used to calculate the relative expression levels. The tubulin gene was selected as an internal reference to normalize the expression of the target gene. Error bars represent Standard Deviation of triplicate experiments and letters above the bars denote significant difference at P<0.05(Turkey’s test).

## Slide 2
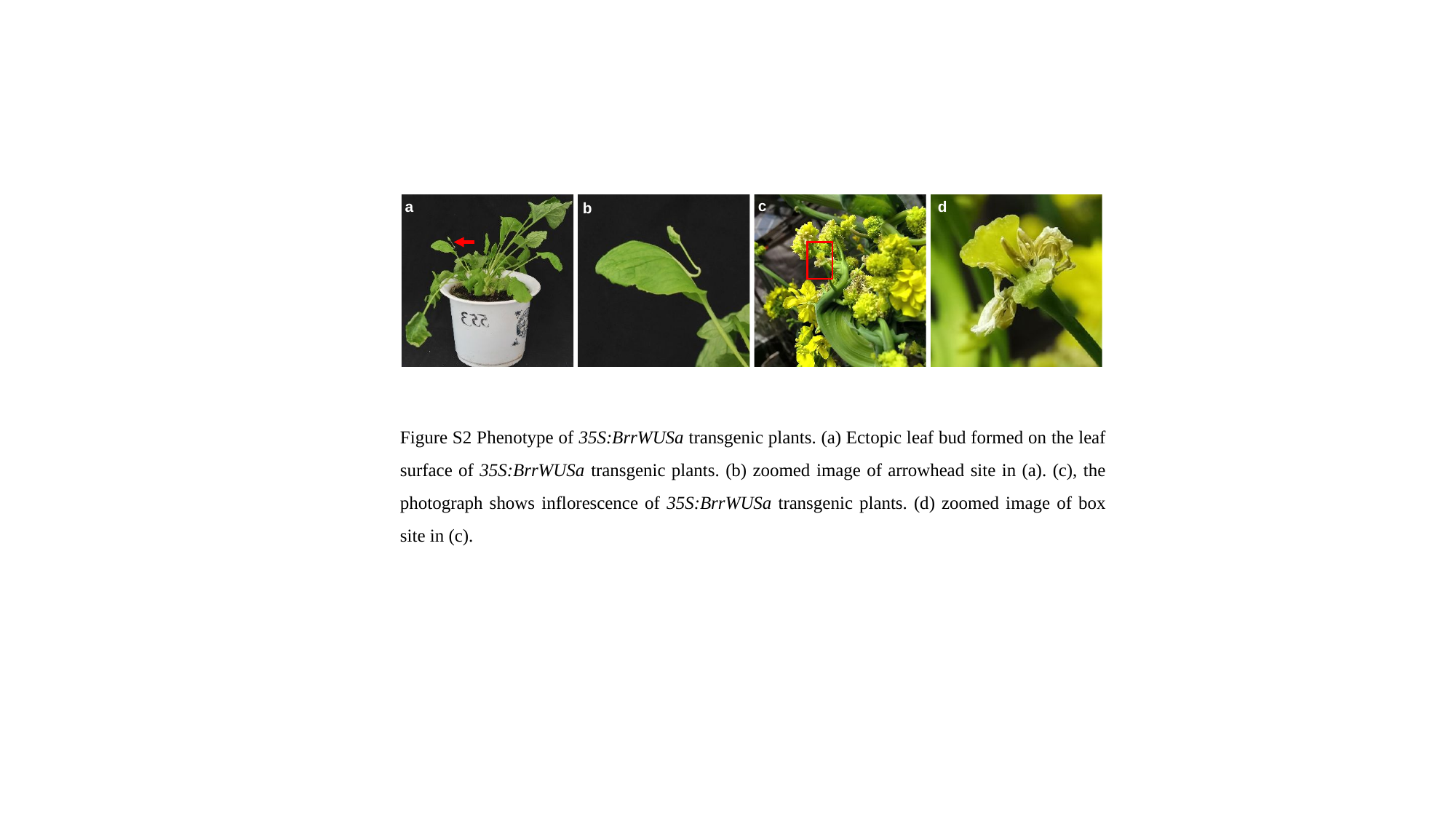

c
d
a
b
Figure S2 Phenotype of 35S:BrrWUSa transgenic plants. (a) Ectopic leaf bud formed on the leaf surface of 35S:BrrWUSa transgenic plants. (b) zoomed image of arrowhead site in (a). (c), the photograph shows inflorescence of 35S:BrrWUSa transgenic plants. (d) zoomed image of box site in (c).

## Slide 3
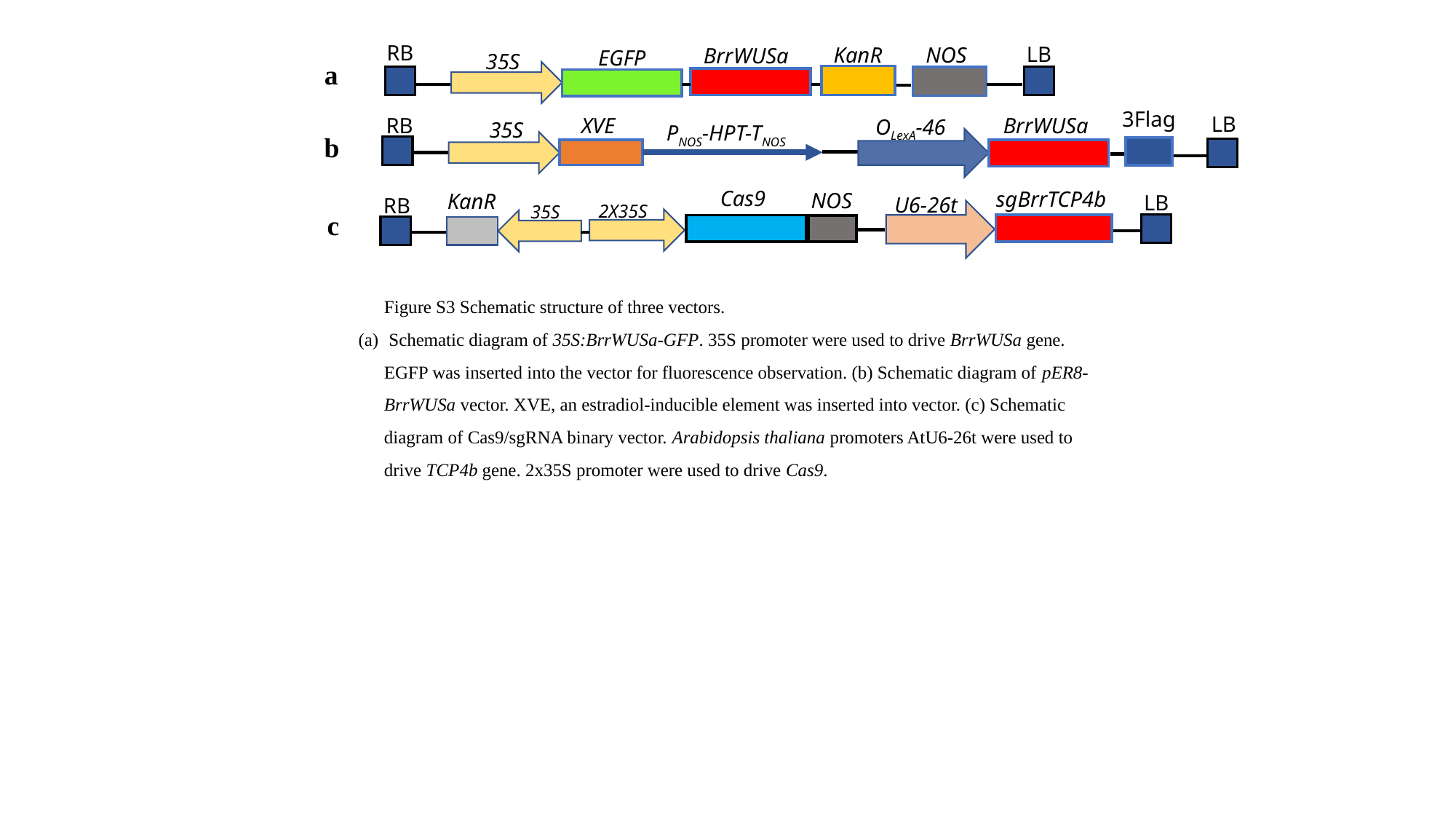

RB
LB
KanR
NOS
BrrWUSa
EGFP
35S
a
3Flag
LB
RB
XVE
BrrWUSa
OLexA-46
35S
PNOS-HPT-TNOS
b
Cas9
sgBrrTCP4b
NOS
KanR
LB
U6-26t
RB
2X35S
35S
c
 Figure S3 Schematic structure of three vectors.
 Schematic diagram of 35S:BrrWUSa-GFP. 35S promoter were used to drive BrrWUSa gene. EGFP was inserted into the vector for fluorescence observation. (b) Schematic diagram of pER8-BrrWUSa vector. XVE, an estradiol-inducible element was inserted into vector. (c) Schematic diagram of Cas9/sgRNA binary vector. Arabidopsis thaliana promoters AtU6-26t were used to drive TCP4b gene. 2x35S promoter were used to drive Cas9.

## Slide 4
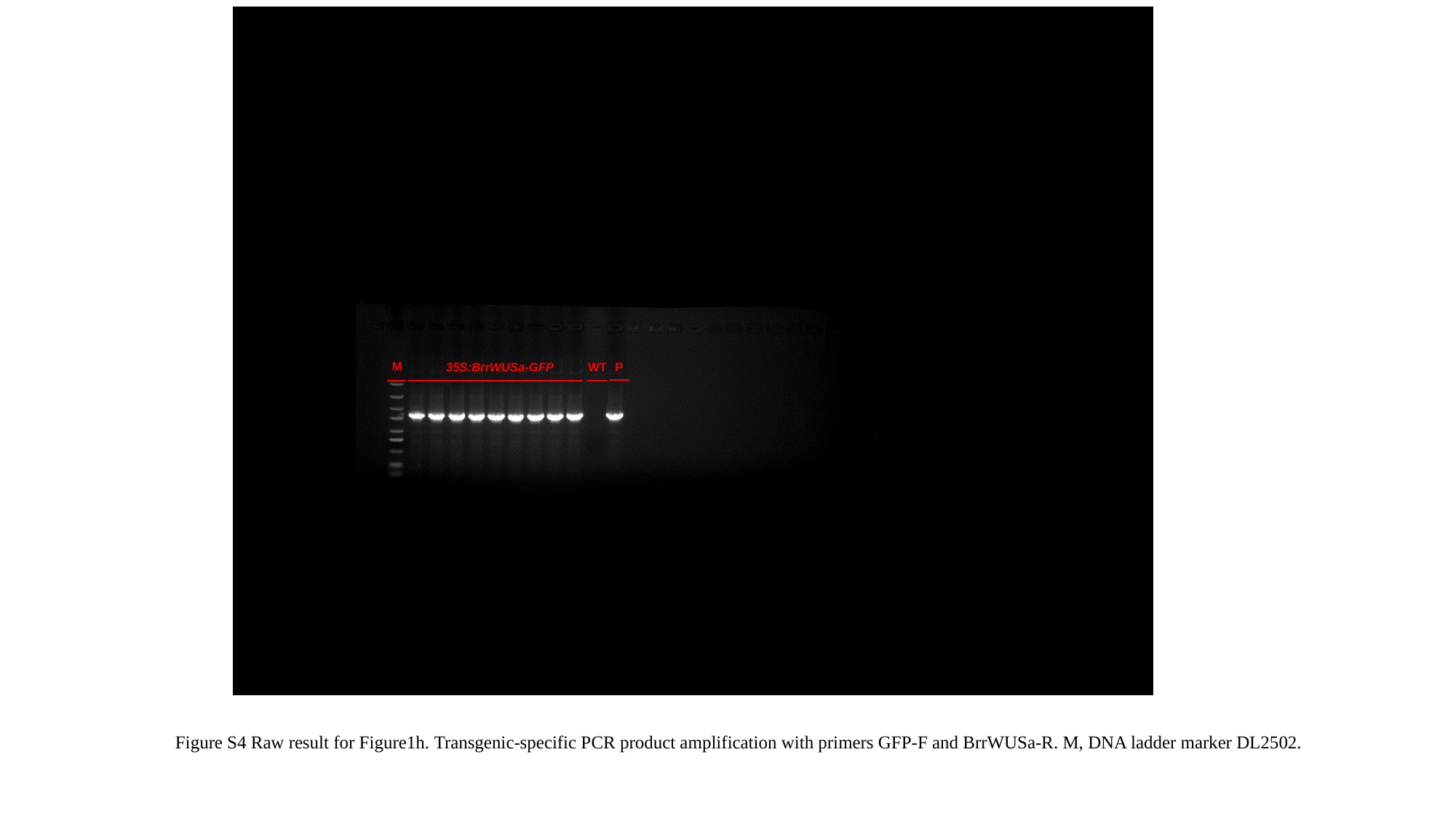

M
35S:BrrWUSa-GFP
WT
P
 Figure S4 Raw result for Figure1h. Transgenic-specific PCR product amplification with primers GFP-F and BrrWUSa-R. M, DNA ladder marker DL2502.

## Slide 5
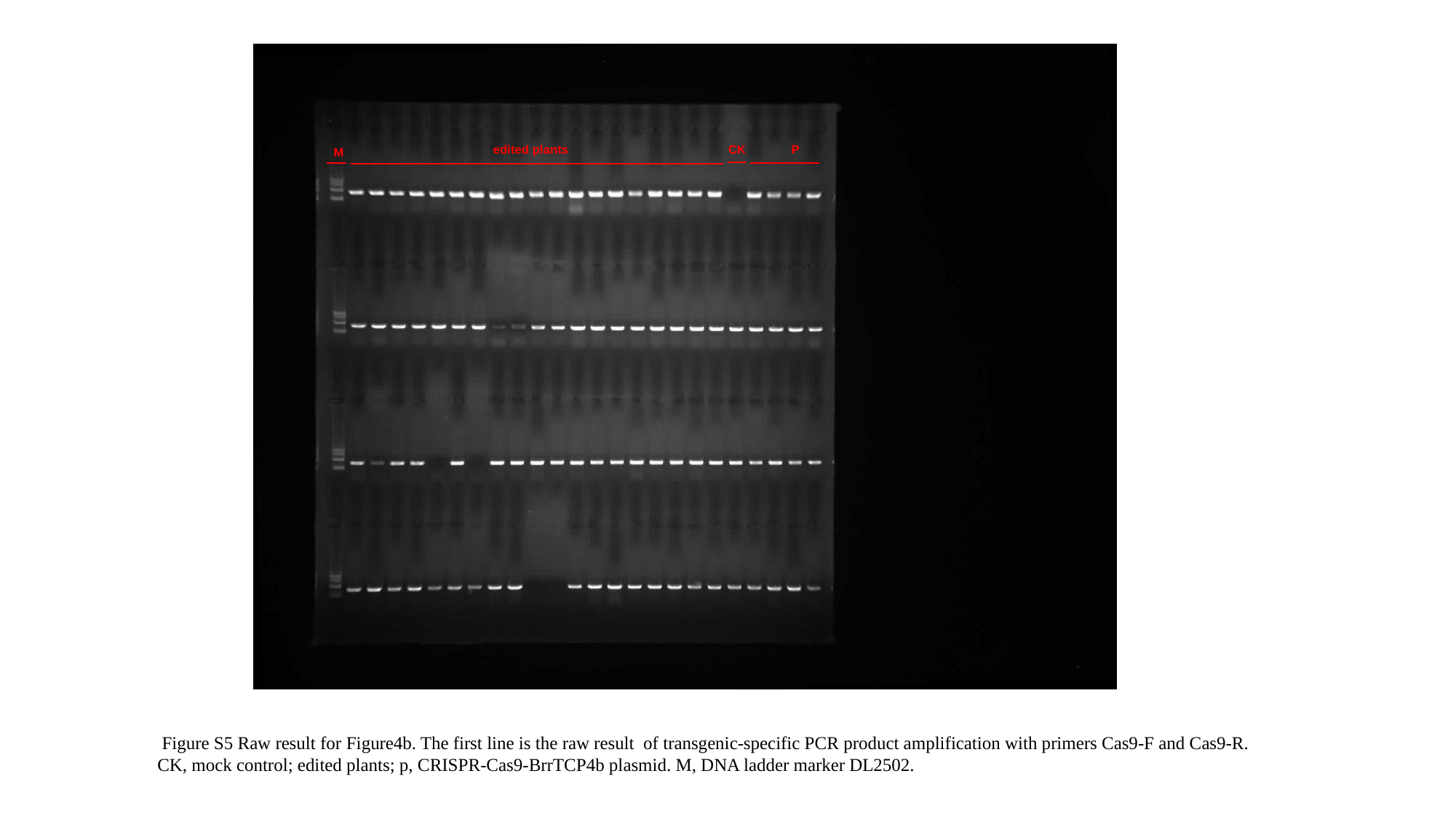

edited plants
CK
P
M
 Figure S5 Raw result for Figure4b. The first line is the raw result of transgenic-specific PCR product amplification with primers Cas9-F and Cas9-R.
CK, mock control; edited plants; p, CRISPR-Cas9-BrrTCP4b plasmid. M, DNA ladder marker DL2502.

## Slide 6
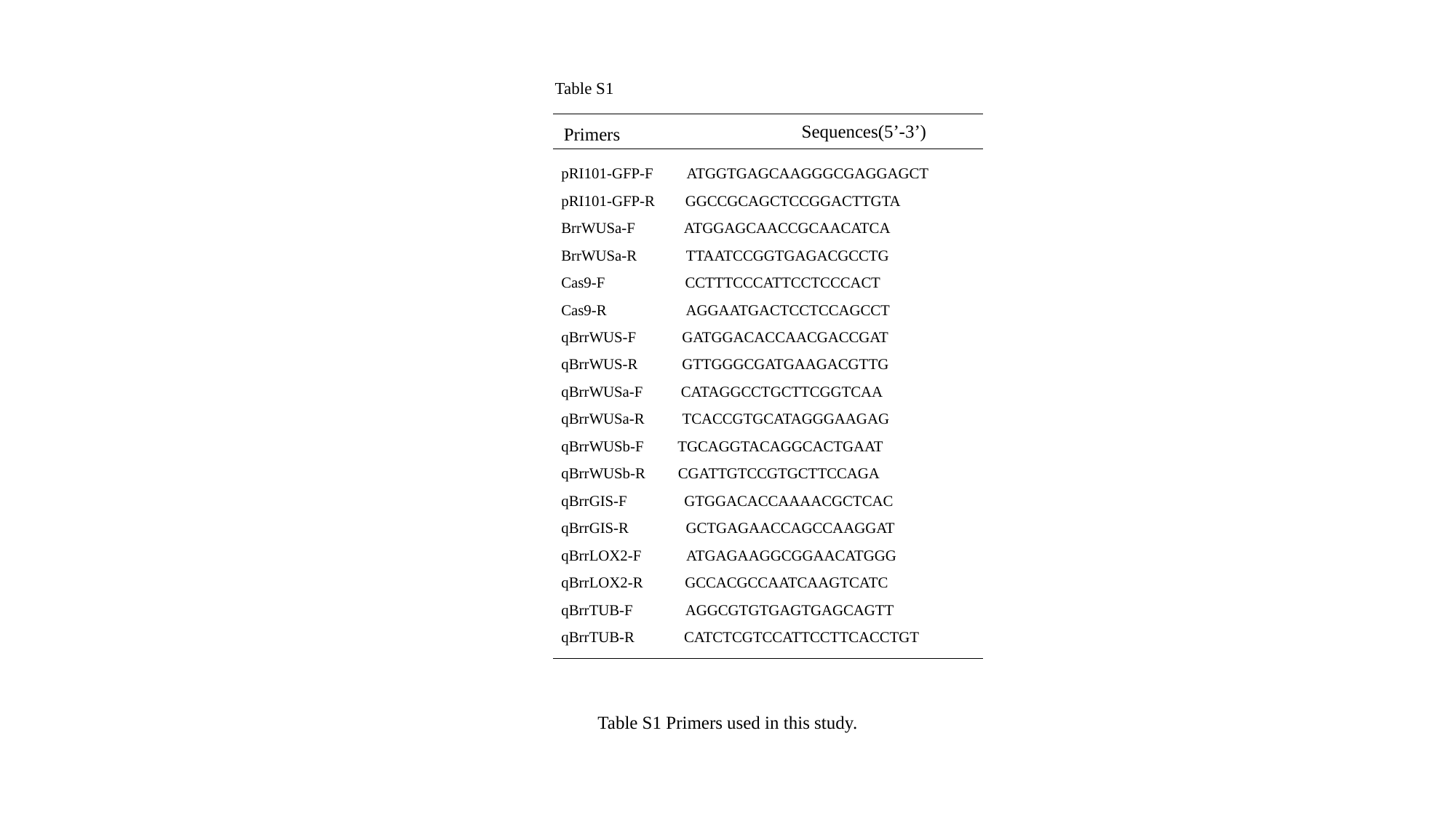

Table S1
Sequences(5’-3’)
Primers
pRI101-GFP-F ATGGTGAGCAAGGGCGAGGAGCT
pRI101-GFP-R GGCCGCAGCTCCGGACTTGTA
BrrWUSa-F ATGGAGCAACCGCAACATCA
BrrWUSa-R TTAATCCGGTGAGACGCCTG
Cas9-F CCTTTCCCATTCCTCCCACT
Cas9-R AGGAATGACTCCTCCAGCCT
qBrrWUS-F	 GATGGACACCAACGACCGAT
qBrrWUS-R	 GTTGGGCGATGAAGACGTTG
qBrrWUSa-F CATAGGCCTGCTTCGGTCAA
qBrrWUSa-R TCACCGTGCATAGGGAAGAG
qBrrWUSb-F	 TGCAGGTACAGGCACTGAAT
qBrrWUSb-R	 CGATTGTCCGTGCTTCCAGA
qBrrGIS-F GTGGACACCAAAACGCTCAC
qBrrGIS-R GCTGAGAACCAGCCAAGGAT
qBrrLOX2-F ATGAGAAGGCGGAACATGGG
qBrrLOX2-R GCCACGCCAATCAAGTCATC
qBrrTUB-F AGGCGTGTGAGTGAGCAGTT
qBrrTUB-R CATCTCGTCCATTCCTTCACCTGT
Table S1 Primers used in this study.
